# The development of compulsive coping behaviour is associated with a downregulation of Arc in a Locus Coeruleus neuronal ensemble

**DOI:** 10.1038/s41386-022-01522-y

**Published:** 2023-01-12

**Authors:** Clara Velazquez-Sanchez, Leila Muresan, Lucia Marti-Prats, David Belin

**Affiliations:** 1 CLIC (Cambridge Laboratory for research on Impulsive/Compulsive disorders), Department of Psychology, University of Cambridge, Downing Street, Cambridge, CB2 3EB UK; 2 Cambridge Advanced Imaging Centre, Department of Physiology Development and Neuroscience of the University of Cambridge, Downing Street, Cambridge, CB2 3DY UK

**Keywords:** Stress and resilience, Cellular neuroscience

## Abstract

Some compulsive disorders have been considered to stem from the loss of control over coping strategies, such as displacement. However, the cellular mechanisms involved in the acquisition of coping behaviours and their subsequent compulsive manifestation in vulnerable individuals have not been elucidated. Considering the role of the locus coeruleus (LC) noradrenaline-dependent system in stress and related excessive behaviours, we hypothesised that neuroplastic changes in the LC may be associated with the acquisition of an adjunctive polydipsic water drinking, a prototypical displacement behaviour, and the ensuing development of compulsion in vulnerable individuals. Thus, male Sprague Dawley rats were characterised for their tendency, or not, to develop compulsive polydipsic drinking in a schedule-induced polydipsia (SIP) procedure before their fresh brains were harvested. A new quantification tool for RNAscope assays revealed that the development of compulsive adjunctive behaviour was associated with a low mRNA copy number of the plasticity marker Arc in the LC which appeared to be driven by specific adaptations in an ensemble of tyrosine hydroxylase (TH)+, zif268− neurons. This ensemble was specifically engaged by the expression of compulsive adjunctive behaviour, not by stress, because its functional recruitment was not observed in individuals that no longer had access to the water bottle before sacrifice, while it consistently correlated with the levels of polydipsic water drinking only when it had become compulsive. Together these findings suggest that downregulation of Arc mRNA levels in a population of a TH+/zif268− LC neurons represents a signature of the tendency to develop compulsive coping behaviours.

## Introduction

Failure adaptively to regulate emotions, such as coping with stress, has been associated with an individual vulnerability to develop several neuropsychiatric disorders, such as anxiety, depression, post-traumatic stress disorder, as well as Impulsive/Compulsive Spectrum Disorders (ICSDs), including obsessive compulsive (OCD) and substance use disorder [1–4]. The emergence of compulsion, which characterises the excessive and persistent nature of several ICSDs [5] has been suggested to stem from a loss of control over coping strategies [6–15], such as displacement behaviours, which initially aim to decrease negative affect or stress in adverse situations but progressively become habitual and rigid [16–18].

Across species, adjunctive behaviours [19] represent a form of displacement activity [20, 21] in which individuals engage to decrease stress [20, 22–24]. One such adjunctive anxiolytic response, schedule-induced polydipsia (SIP) [20, 21, 25–28] is manifested as a non-regulatory polydipsic water drinking in the face of intermittent food delivery in food-restricted animals [28–31]. Adjunctive drinking in SIP transiently decreases autonomic nervous system responses to stress [32] and the plasma levels of stress-related hormones such as corticosterone [21, 25–27, 32–36] which are elevated by chronic exposure to intermittent food delivery [37] and necessary for its full development [34]. This transient decrement in plasma level of stress hormones following exposure to SIP is not observed when individuals do not have access to water [25].

Like in humans, the majority of individuals who engage in displacement behaviours as a coping strategy tend, at the population level, to maintain relative control over their adjunctive response [18, 38–41]. Nevertheless, some vulnerable individuals, characterised, for instance, by a high impulsivity trait [40], lose control over their polydipsic intake, which becomes excessive and inflexible [18, 38–43], thereby suggesting that poor impulse control prior to the engagement in an adjunctive behaviour facilitates the transition to compulsivity [40].

The psychological and neural basis of the individual vulnerability to develop such compulsive adjunctive behaviours has not been fully elucidated. Since the demonstration that the development of SIP is prevented by 6-OHDA lesions of the mesolimbic system [44] and its compulsive manifestation is decreased by selective serotonin reuptake inhibitors [for review, see 43], research into the neural systems basis of compulsive adjunctive behaviours has primarily focused on dopaminergic and serotoninergic mechanisms within the corticostriatal circuitry [42, 43, 45]. However, increasing evidence supports a role of the noradrenergic system in compulsive adjunctive behaviours [41, 46] not least because of the involvement of noradrenergic mechanisms in the regulation of glucocorticoids levels [47, 48] and the stress-induced repetitive behaviours [49], but also because of their role in anxiety, coping and impulse control [50–53], which all contribute to the emergence and the severity of compulsions [54–56].

In SIP, the emergence of compulsive adjunctive drinking is also associated with higher levels of noradrenaline (NA) in the amygdala and the nucleus accumbens (Nac) [57] in which elevated levels of NA are also observed in impulsive, SIP-prone rats [58]. In addition, in highly impulsive rats, intra-accumbens shell (NacS) infusions of the noradrenaline reuptake inhibitor atomoxetine decrease impulsivity similarly to its systemic administration [59], which also prevents the development of compulsive adjunctive drinking under SIP in these animals [41].

While the NacS receives noradrenergic inputs from the A2 noradrenergic population of the Nucleus of the Solitary tract (NTS) and the Locus Coeruleus (LC) [60], the acquisition of coping responses has been associated with the LC-NA system [61–65] which has also been suggested to be involved in the expression of polydipsic adjunctive water drinking [66].

Together, these observations suggest that specific adaptations taking place in the LC may contribute to the noradrenergic mechanisms, especially in the NacS, that subserve impulsivity and the associated vulnerability to develop compulsive adjunctive behaviours. However, the high functional and cytoarchitectural heterogeneity of the LC [67, 68] with subdivisions or particular neuronal ensembles [69, 70] or microcircuits [71] involved in different aspects of behaviour [71–73], including stress-related mechanisms [74], warrants a mapping of its projections to the NacS.

At the cellular level, the response of the LC-NA system to a variety of stressful situations, including restraint, exposure to mild electric shocks or social stress, has been associated with the recruitment of immediate early genes (IEGs) [75, 76] including activity or plasticity-related transcription factors, such as c-fos or zif268, respectively, and effectors, such as activity-related cytoskeleton-associated protein (Arc), which instead directly influence cellular processes other than gene transcription [77]. Thus, exposure to novelty, anxiogenic drugs, repeated episodes of restraint stress or social stress results in an increase in c-fos in the LC [78–82]. However, c-fos mRNA levels tend to decrease over repeated exposure to stressful situations [83, 84] which precludes its use as an ensemble marker of daily adaptive or compulsive adjunctive responding over several weeks. This is not the case of Arc [76], an effector of BDNF, glutamatergic, dopaminergic and serotoninergic signalling that is involved in learning-associated synaptic and dendritic plasticity [85, 86], and is associated with behavioural abnormalities, including schizophrenia-like symptoms [87]. Arc mRNA levels in the LC have been shown, using standard in-situ hybridisation not to respond to acute stress, but instead to situations of adaptation to chronic challenges [88].

Thus, here we investigated whether the emergence of compulsive adjunctive behaviour was associated with the recruitment of a neuronal ensemble that is characterised by a selective engagement or Arc-dependent cellular plasticity in a territory of the LC that projects to the region of the NacS mediating noradrenergic influence over impulse control. Because Arc transcription is under the control of zif268, even though their mRNA levels do not necessarily correlate [89], we sought to determine whether any potential Arc-dependent ensemble was specifically engaging Arc or whether it was also reflecting the activation of zif268. For this, we developed a new RNAscope multiplex assay quantification method in order to investigate the mRNA copies in genetically identified cellular ensembles in the LC of rats with controlled or compulsive adjunctive behaviour sacrificed 45 min after a challenge SIP session during which they expressed their polydipsic drinking behaviour or were prevented from doing so.

## Methods and materials

### Subjects

Two independent experiments were carried out that each involved forty-eight male Sprague Dawley rats (Charles River, UK) weighing approximately 300 g at the start of the experiment and were single-housed under a reversed 12 h light/dark chain (lights off at 7:00 a.m.). After a week of habituation to the vivarium, rats were food restricted to gradually reach 80% of their theoretical free-feeding body weight before starting behavioural training. Water was always available *ad libitum*. Experiments were performed 6–7 days/week between 8 a.m. to 5 p.m. All experimental protocols were conducted under the project license 70/8072 held by David Belin in accordance with the regulatory requirement of the UK Animals (Scientific Procedures) Act 1986, amendment regulations 2012, following ethical review by the University of Cambridge Animal Welfare and Ethical Review Body (AWERB).

### Timeline of the experiments

**Fig. 1:**
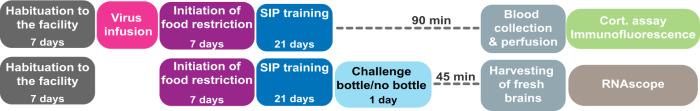
Timeline of the experiments. Two independent experiments were carried out in the present study that involved forty-eight male Sprague Dawley rats each. After 1 week of habituation to the animal facility, rats involved in the first experiment received intra-accumbens shell (NacS) infusion of a retrograde CAV2-GFP virus under stereotaxic surgery and were left undisturbed for at least one week. Rats from both experiments were progressively food restricted to 80% of their theoretical free-feeding body weight. Then, following two sessions of habituation to the SIP context during which their regulatory water intake was measured, rats were trained under a Fixed time (FT) 60s Scheduled-Induced polydipsia (SIP) procedure for 21 1 h daily sessions. High drinkers (HD) and Low drinkers (LD) rats were selected in the upper and lower quartile of the population, respectively, based on their average water intake during the last 3 sessions. Ninety minutes after the last SIP session, rats from experiment 1 underwent a blood collection prior to being perfused transcardiacally in order for their brains to be processed for immunofluorescence. In contrast, rats from experiment 2 were sacrificed forty-five minutes after a 60 min challenge session with or without the opportunity to express their adjunctive behaviour, and their fresh brains were harvested and properly stored subsequently to be used for RNAscope assays. Exp. experiment. Cort. corticosterone.

The timeline of the experiments is illustrated in Fig. 1 and detailed in the Supplement Online Methods SOM. Briefly, after one week of habituation to the vivarium, rats in experiment 1 received intra-NacS infusion of a retrograde CAV2-GFP virus under stereotaxic surgery. Then, rats in both experiments were progressively food restricted to 80% of their theoretical free-feeding body weight. They were accustomed to the SIP context over two habituation sessions during which their regulatory water intake was measured, and then trained in a SIP procedure for 21 daily sessions. Ninety minutes after the last SIP session, rats in experiment 1 underwent a blood collection, in order to assess post-SIP plasma corticosterone levels, after what they were perfused transcardiacally, and their brains were harvested in order subsequently to map the projections of the LC to the NacS. Rats from experiment 2 were sacrificed 45 min after a challenge session with or without the opportunity to express their adjunctive behaviour, and their fresh brains were harvested and subsequently used in RNAscope assays.

### Apparatus

The SIP procedure was carried out in 12 operant chambers (Med Associates Inc., Ltd) as previously described [41, 90] and detailed in the SOM.

#### Schedule-induced polydipsia (SIP)

The SIP procedure based on a fixed-time 60-s schedule of food delivery was carried out as previously described [38, 41, 43, 90] and detailed in the SOM.

Water intake over the last 3 sessions was used to identify rats in the upper and lower quartile of the population as High drinkers (HD, *n* = 14) and Low drinkers (LD, *n* = 14), respectively, as previously described [41, 91].

Subsequently, in order to establish whether the cellular correlates of the tendency to develop compulsive polydipsic behaviour were attributable specifically to the expression of the anxiolytic adjunctive response, or instead to the distress induced by the procedure, on day 22 rats from experiment 2 underwent one 1 h challenge SIP session during which half the population had access to the bottle of water (*n* = 24) and could express their adjunctive response or had their water bottle removed (*n* = 24), thereby being prevented from engaging in their well-established coping habit [18], which we speculated should result in negative urgency [92] associated with heightened stress and frustration [23, 93].

### Experiment 1

#### Stereotaxic surgery and viral infusions

In order to identify which territory of the LC projects to the area of the NacS in which infusions of atomoxetine recapitulate the effect of its systemic administration on impulse control [59] intra-NacS infusions of a CAV2-GFP virus were carried out unilaterally at 4 different anteroposterior coordinates (Fig. 3) using a stereotaxic frame (WPI Hitchin, UK) under isoflurane anaesthesia (O_2_: 2 L/min; 5% for induction and 2–3% for maintenance) and analgesia (Metacam, 1 mg/kg, sc., Boehringer Ingelheim). The analgesic treatment was continued orally for three days post-surgery. The forty-eight rats were divided into four groups, each receiving a unilateral CAV2-Cre virus (109 vp/μl, 1 μl/side) infusion at the following stereotaxic coordinates AP:+2.76, +2.28, +1.7 or +1.08; ML: ±1.0, ±1.0, ±0.8, ±1.0; DV: − 6.8, −7.2, −7.25, −7.1, respectively (from the skull) [94]. Infusions were performed at a rate of 0.15 μl/min with a 10 ul Hamilton syringe placed in a Harvard infusion pump and connected with a polyethylene tubing to 24-gauge injectors (Coopers needle works Ltd). Injectors were left in place for 7 min after the infusion to allow for diffusion. Animals were sacrificed at least 2 months after the viral infusion, so that the retrograde virus had time to travel from the injection site to the LC.

#### Histology

As described in detail in the SOM each perfused brain was harvested, cryoprotected and frozen before being processed into 30-μm-thick coronal sections using a cryostat (Leica CM3050 S Research Cryostat) and stored in a cryoprotectant solution at −20 °C until being processed for immunofluorescence.

#### GFP immunofluorescence

Sections ranging from −9.6 to −9.96 mm from bregma, a rostrocaudal region that entirely encompasses the LC were processed for immunofluorescence with a chicken anti-GFP (1:1000; abcam, ab13970) primary antibody and a goat anti-chicken (AF488, 1:1000; ThermoFisher Scientific, A-11039) secondary antibody prior to being mounted onto glass slides (Fisherbrand Superfrost Microscope Slides), allowed to dry overnight (protected from light) and covered with a coverslip and fluoroshield mounting medium (abcam, ab104135). See SOM for a detailed description of the procedure.

Images were acquired with a Zeiss Axio Imager M2 equipped with an AxioCam MRm camera (Oberkochen, Germany) using Visiopharm® software (Medicon Valley, Denmark), either at x5 magnification and tiled to create the whole slice images or at  x10 magnification for a detailed analysis of the region of interest, namely the LC.

#### Corticosterone assay

The quantification of the post-SIP plasma corticosterone level of HD and LD rats of experiment 1 was carried out by ELISA on samples collected 90 min after a SIP session according to the manufacturer’s instruction (Cayman Chemical, 501320) as detailed in the SOM.

### Experiment 2

#### Histology

Forty-five minutes after the challenge session, the brain of each individual was harvested fresh after decapitation, snap frozen at −40 °C in isopentane (Sigma-Aldrich) and stored at −80 °C, as previously described [92] and detailed in the SOM. Brains were then processed using a cryostat (Leica Microsystems) into 12-μm-thick coronal sections collected on Superfrost gelatine-coated slides (Fisher Scientific) and stored at −80 °C until they were processed for multiplex RNAscope® in situ hybridisation.

#### RNAscope® in situ hybridisation assay

RNAscope was performed according to the manufacturer’s instructions for fresh frozen tissue using the RNAscope Multiplex Fluorescent Reagent Kit (Advanced Cell Diagnostics), as detailed in the SOM.

#### RNAscope® in situ hybridisation imaging and quantification

Images for quantitative RNAscope analysis were captured with a Zeiss Axio Imager M2 equipped with an AxioCam MRm camera (Oberkochen, Germany) using Visiopharm® software (Medicon Valley, Denmark) using a 63× objective oil immersion lens. For each rat and RNAscope assay, 8 images spanning the entire rostrocaudal axis of the LC, ranging from −9.60 to −9.96 mm AP relative to Bregma [94], were analysed (Fig. 3C).

Although RNAscope has recently gained popularity and is now widely used, the quantification strategy of the signal it yields has hitherto been sub-optimal, being limited to either the measurement of the signal intensity per channel/wavelength/probe or the quantification of mRNA positive cells, thereby missing out on the opportunity to systematically quantify the number of mRNA molecules on a cell or a structure.

Several methods using semi-quantitative and quantitative analysis have been developed [95, 96], but they too often rely on algorithms that require highly specialised coding skills, rendering them difficult to use by the many laboratories that do not have such expertise. In addition, most of the microscopy-based quantification methods and softwares available through different commercial platforms are semi-quantitative approaches that do not make full use of the single molecule quantification opportunity given by RNAscope. Thus, in order to maximise the information generated by the multiplex RNAscope assay, we developed a new analysis pipeline that goes beyond a simple quantification of light intensity to assess the levels of a target mRNA.

Our RNAscope signal analysis pipeline relies on a MATLAB (MATLAB - R2020a, The MathWorks Inc) script combined with a machine learning algorithm that uses ImageJ/FIJI (National Institutes of Health, Bethesda, MD, USA) to identify a single cell-delineated region of interest (ROI) based on a segmentation on the DAPI signal and the ensuing determination of an area (the ROI) around it within which to count single mRNA molecules.

mRNA molecules appear in images as bright “*dots*”, those with intensity exceeding chosen thresholds are considered true detections. For the detection of single mRNA molecules corresponding to the relevant channels 1, 2 and 4 the script enables the choice of independent threshold parameters (th1, th2 and th4). In order to remove the background and maximise the signal-to-noise ratio, a difference of Gaussian filtering pre-processing step is applied. The raw image is blurred by convolution with a small Gaussian, σ1 = 0.5 pixels, to enhance signal. The background estimated by a larger Gaussian filtering, σ2 = 3 pixels, is subsequently subtracted from the image.

Nuclei segmentation is performed on the DAPI (third) channel. The pipeline allows the selection of a minimum area for nucleus detection. A Gaussian filter (σ = 7 pixels) is applied to the DAPI image in order to smooth intensity inhomogeneities. Subsequently, a multi threshold quantisation is performed, detecting three levels of nuclei intensities, since not all the DAPIs (or ROIs) are equally bright due to a different location along the Z-axis. Touching nuclei are separated via a watershed transformation (adapted to the levels of intensities detected in the image). Additionally, and as an optional step, all the DAPI segmentations can be manually corrected using the imageLabeler app in MATLAB.

However, watershed techniques are very susceptible to over-splitting and become less accurate in the case of elliptical cells and can be prone to segmentation errors if the nuclei are densely clustered together. In order to overcome these issues, we alternatively used StarDist [97] (in ImageJ/FIJI) for nuclei segmentation. StarDist is a segmentation method based on a deep learning, U-Net architecture that localises cell nuclei approximated as star-convex polygons.

Once all the parameters have been set based on a sample image, and the detection of the different mRNAs and cell nuclei is deemed appropriate by the experimenter, they are kept unchanged for the entire RNAscope assay or batch image analysis.

Finally, all the processed images pertaining to the same experiment are analysed, mRNA molecules closer than a certain distance (10 pixels) to a nucleus are assumed pertaining to that nucleus. The detections in each channel corresponding to each ROI are summarised and exported in Excel files.

The software package is available at https://gitlab.com/lemur01/rnascopeanalysis.

### Data and statistical analyses

Data presented as means ± SEM or box plots [medians ±25% (percentiles) and Min/Max as whiskers] were analysed using STATISTICA 10 software (Statsoft, Palo Alto). Assumptions for normal distribution, homogeneity of variance and sphericity were confirmed using the Shapiro–Wilk, Levene, and Mauchly sphericity tests, respectively.

The nature of the adjunctive drinking behaviour of each individual was characterised at the end of the procedure, based on the average water drinking behaviour across the last three sessions as being compulsive (HD rats, upper quartile), intermediate (intermediate rats) or low (LD rats, lower quartile). For experiment 2, differences in performance between the individuals that would have the bottle or the bottle removed during test were analysed independently for the HD and LD groups using a Student’s *t*-test.

Water intake during the daily sessions of the SIP procedure was analysed using a repeated-measure analysis of variance (ANOVA) with sessions as within‐subject factor and phenotype (High and Low drinkers; HD, LD) and Challenge (Bottle and no bottle; B, NB, only for experiment 2) as between-subject factors. Upon confirmation of significant main effects, differences were analysed using the Newman-Keuls post hoc test.

Since only 20 slides can be processed in the same RNAscope assay, considering the high signal/noise ratio and sensitivity of the technique, only five representative individuals of the HD and LD subpopulations were used for RNAscope. One animal belonging to the HD-B group was removed from the zif268-TH-Arc RNAscope data due to tissue damage during the assay so that the final samples sizes are HD-NB: *n* = 5, HD-B: *n* = 4, LD-NB: n = 5, LD-B: *n* = 5.

RNAscope images data belonging to the same animal were summed and then averaged across groups. The Kruskal-Wallis test was used to compare the different mRNA levels in the LC from HD and LD rats. Relationships between mRNA levels and water intake during SIP sessions were investigated using Spearman correlations and the *p* values obtained were subsequently corrected for multiple comparisons by the Benjamini-Hochberg method [98, 99].

Differences in plasma corticosterone concentrations between HD (*n* = 11) and LD (*n* = 7) individuals randomly selected from the upper and lower quartile of the population were analysed using a Student’s *t*-test.

For all analyses, significance was set at *α* = 0.05. Effect sizes are reported as partial eta squared (p*η*^2^).

## Results

**Fig. 2:**
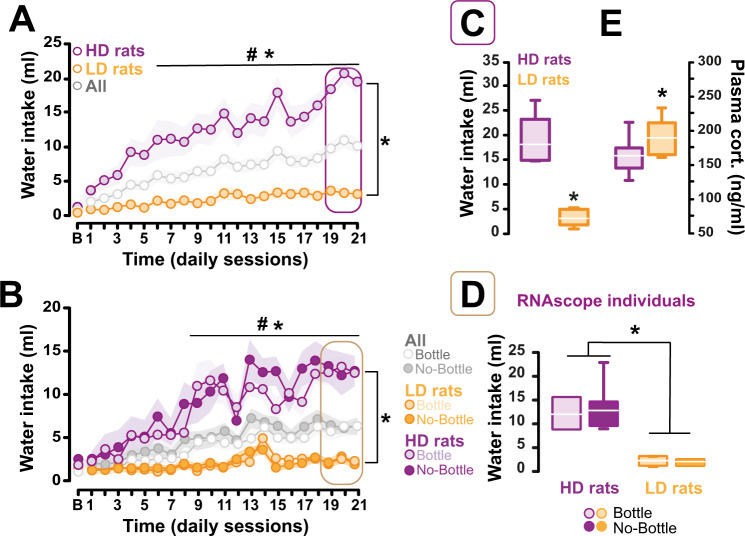
Characterisation of the individual vulnerability to develop compulsive adjunctive behaviour under a Schedule-Induced Polydipsia (SIP) procedure. The two cohorts exposed to a SIP procedure in experiment 1 **(A)** and 2 **(B)** developed a similar polydipsic adjunctive water drinking behaviour over the course of 21 daily sessions. In both cohorts, marked individual differences were observed in the tendency to develop compulsive adjunctive behaviour. HD rats, whose daily water drinking behaviour started to differ from that of LD rats and their own baseline drinking after 6 and 8 sessions (for experiment 1 and 2, respectively) eventually drank, by the end of training, more than 3 times as much water as LD rats, whose drinking behaviour never differed from baseline [main effect of phenotype: F_1,26_ = 118.143, *p* > 0.001, pη^2^ = 0.819 and F_1,16_ = 45.76, *p* ≤ 0.001, pη^2^ = 0.74 for experiment 1 and 2, respectively] (**C**, **D**). The compulsive adjunctive behaviour shown by HD (*n* = 11) rats resulted in a decrease in plasma corticosterone after a SIP session as compared to LD rats (*n* = 7) [*t* = 2.27 *p* = 0.036] (**E**). The LD (LD-NB *n* = 5, LD-B *n* = 5) and HD individuals (HD-NB *n* = 5, HD-B *n* = 4) that were assigned to the bottle or no-bottle condition during the challenge session in experiment 2 displayed similar levels of adjunctive drinking [*t* = 0.12 *p* = 0.90 and *t* = −0.22, *p* = 0.82, respectively] (**D**). * Different from LD rats; *p*s ≤ 0.05. # HD rats: different from baseline; *p*s ≤ 0.001.

The two cohorts exposed to a SIP procedure in experiment 1 and 2 developed a similar polydipsic adjunctive water drinking behaviour over the course of 21 daily sessions [main effect of session: *F*_21,987_ = 28.702, *p* ≤ 0.001, pη^2^ = 0.379 and *F*_20,940_ = 22.65, *p* ≤ 0.001, pη^2^ = 0.325 for experiment 1 and 2, respectively] (Fig. 2A, B), thereby demonstrating that the expression of a transgene in the NacS-projecting LC neurons does not interfere with the development of this adjunctive behaviour.

In line with previous reports [41, 91], in both cohorts, marked individual differences were observed in the tendency to develop compulsive adjunctive behaviour [main effect phenotype: *F*_1,26_ = 39.344, *p* ≤ 0.001, pη^2^ = 0.602, and *F*_1,24_ = 58.623, *p* ≤ 0.001, pη^2^ = 0.70 and phenotype x session interaction: *F*_21,546_ = 9.95, *p* ≤ 0.001, pη^2^ = 0.276 and *F*_21,504_ = 12.015, *p* ≤ 0.001, pη^2^ = 0.33, for experiment 1 and 2, respectively]. HD rats, whose coping water drinking differed from that of LD rats and their own baseline from sessions 6 and 8 onwards (for experiment 1 and experiment 2, respectively), eventually drank, by the end of training, more than 3 times as much water as LD rats, the performance of which never differed from baseline [main effect of phenotype: *F*_1,26_ = 118.143, *p* > 0.001, pη^2^ = 0.819 and *F*_1,16_ = 45.76, *p* ≤ 0.001, pη^2^ = 0.74 for experiment 1 and 2, respectively] (Fig. 2C, D). At this point, the compulsive adjunctive behaviour shown by HD rats still resulted in an acute decrease in plasma corticosterone levels after SIP as compared to LD rats [*t* = 2.27 *p* = 0.036] (Fig. 2E).

The LD and HD individuals that were assigned to the bottle or no-bottle condition during the challenge session in experiment 2 displayed similar levels of adjunctive drinking [*t* = 0.12 *p* = 0.90 and *t* = −0.22, *p* = 0.82, respectively] (Fig. 2D).

**Fig. 3:**
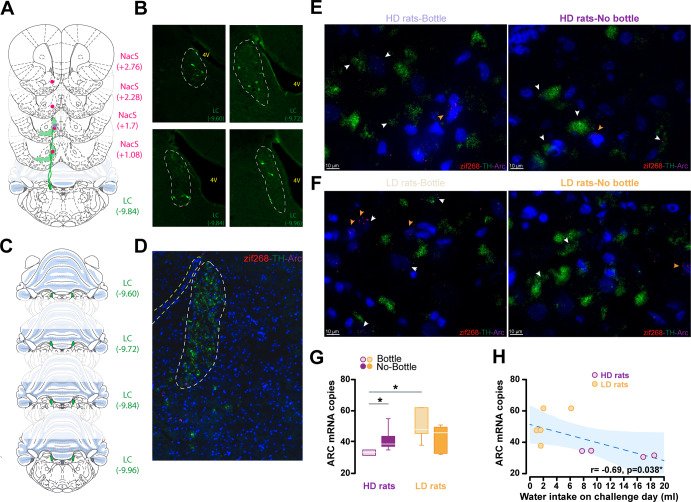
The expression of compulsive adjunctive behaviour is associated with a decrease in Arc mRNA levels across the Locus Coeruleus. **A** Schematic representation of the unilateral infusion sites (pink dots) of a CAV2-GFP retrograde virus into the medial NacS at four different anteroposterior levels (relative to Bregma, coordinates shown next to each brain atlas template extracted from the Paxinos & Watson’s rat brain atlas [94]) encompassing the target territory in which infusion of atomoxetine was shown to recapitulate the effect systemic administration of this noradrenaline reuptake inhibitor on impulse control (blue dot). **B** While labelling of noradrenergic neurons in the A2 nucleus was never observed, infusions in the target territory or adjacent to it caudally, but not in other areas of the NacS resulted in GFP immunofluorescence positive cells across the LC. **C** Schematic representation of the distribution of the coronal sections of the LC ranging from −9.60 to −9.96 (relative to Bregma) that have been processed with RNAscope assays systematically to quantify the number of copies of target mRNAs across the entire LC, as informed by the outcome of the tracing experiment (**A, B**). **D** Representative superimage of a 12μm-thick coronal section, taken at ×40 magnification showing the LC, where cell bodies of tyrosine hydroxylase expressing cells are located, delineated in white and a yellow outline of the edge of the fourth ventricle as anatomical reference, processed with RNAscope targeting the tyrosine hydroxylase, zif268 and Arc mRNAs. Representative 63x images of the LC from HD (**E**) and LD (**F**) rats that had, or not, the opportunity to express their adjunctive polydipsic behaviour during the challenge session that preceded the harvesting of the brains, with the same green, red and magenta, fluorescent probes as in (**D**) targeting TH, zif268 and Arc mRNA, respectively. The white and orange arrows point the presence of Arc and zif268 mRNA molecules, respectively. **G** The individual tendency to eventually express compulsive adjunctive polydipsic drinking behaviour was associated with a lower level of Arc mRNA expression in the LC in that HD rats showed fewer ARC mRNA copies in this monoaminergic nucleus than LD rats. Preventing these HD rats from expressing their compulsive adjunctive polydipsic drinking behaviour by removing their bottle resulted in an increase of Arc mRNA copies in the LC, an effect not observed in LD rats. **H** At the population level, the number of Arc mRNA copies in the LC was found to be correlated with the level of polydipsic drinking during the challenge session [Spearman correlation, *r* = −0.692, *p* = 0.038]. (*p < 0.05).

Mapping of the projections to the territory of the NacS in which noradrenergic mechanisms had been shown to influence the high impulsivity trait that confers increased vulnerability to develop compulsive adjunctive behaviour under SIP revealed no afferent from the A2 region of the NTS but a dense innervation from the LC (Fig. 3A). These NacS-projecting LC neurons were found to be distributed throughout the LC with no apparent pattern alongside any of the rostrocaudal, dorso-ventral and medio-lateral axes (Fig. 3B). Such widespread distribution called for an investigation of compulsivity-associated neuronal ensembles throughout the LC in the ten individuals, representative of the HD and LD groups, that were selected for RNAscope assays to measure the mRNA levels of Arc-TH-Egr1/zif268 and Arc-GFAP-Egr1/zif268 (Fig. 3C–F).

The expression of compulsive adjunctive behaviour was associated with a lower level of Arc mRNA expression in the LC. Thus, HD rats showed fewer Arc mRNA copies in the LC than LD rats [Kruskal Wallis H(3) = 8.185, *p* = 0.043; HD-B vs LD-B *p* = 0.027] (Fig. 3G) and the total number of Arc mRNA copies in the LC was negatively correlated to the intensity of polydipsic water drinking during the challenge session [Spearman correlation, *r* = −0.692, *p* = 0.038] (Fig. 3H).

This lower level of Arc mRNA copies in the LC that characterised compulsive drinking in HD rats was specifically associated with the behavioural expression of the compulsion to engage in an excessive adjunctive behaviour because LC Arc mRNA levels were higher in HD rats that were prevented from expressing their polydipsic drinking by removal of the bottle during the challenge session than in those that had the opportunity to express their abnormal coping response [Kruskal Wallis H(1) = 5.07, *p* = 0.0.243; HD-B vs HD-NB *p* = 0.027], a difference that was not observed in LD rats [Kruskal Wallis H(1) = 1.33, *p* = 0.247; LD-B vs LD-NB *p* = 0.25] (Fig. 3G).

**Fig. 4:**
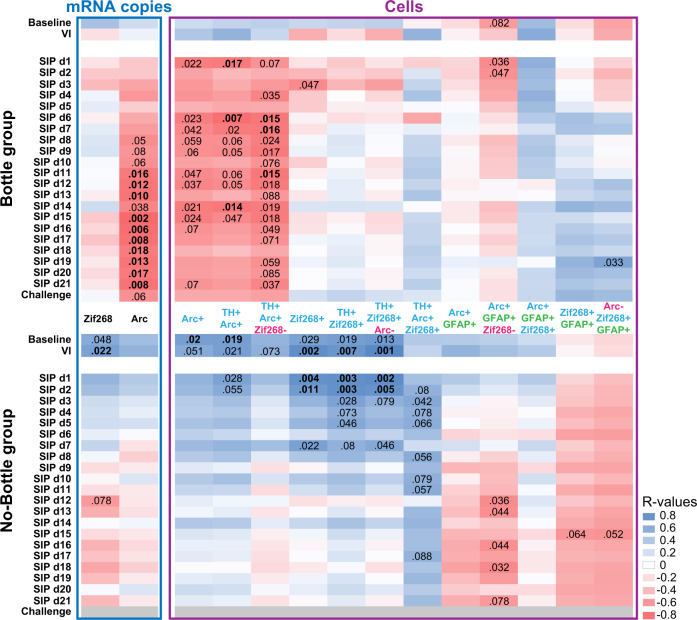
Downregulation of Arc mRNA levels in the LC, especially in TH+ but zif268- cells tracks the development of compulsive adjunctive polydipsic drinking behaviour. The regulation of Arc mRNA levels within the LC by the expression of polydipsic drinking was not uniform across its different cell types and functional ensembles as shown by correlation matrices which track the relationship between the level of polydipsic drinking and (i) the total mRNA copies of zif268 and Arc in the LC and (ii) the representativity of various cell types characterised for their expression of the tyrosine hydroxylase, GFAP, zif268 and Arc, as well as any of their combinations. This dimensional analysis revealed that the negative relationship observed between the number of Arc mRNA copies in the LC and polydipsic drinking only emerged from session 8 onwards, exactly at the time when HD rats started to diverge from LD rats on their route towards the development of compulsive polydipsic drinking. This relationship was not observed for zif268, a distinction suggestive of a differential contribution of the two plasticity markers to the development of compulsive adjunctive behaviour and supported by a very similar pattern of correlation between polydipsic drinking and the percentage number of cells expressing Arc, but not zif268. Further analysis of these matrices revealed that the relationship between polydipsic drinking and Arc mRNA levels was only present in neurons (no correlation was found in GFAP+ cells), more precisely in TH+ neurons and only in those that do not belong to a zif268+ ensemble. Removing the water bottle at test, which prevented rats from expressing their adjunctive polydipsic behaviour, be it compulsive or not, disrupted the correlation with Arc mRNA levels, thereby revealing that the downregulation of Arc in TH+ zif268- cell ensembles tracks the expression of a compulsive adjunctive response.

Further retrospective dimensional analyses revealed that the negative relationship observed between Arc mRNA copy number in the LC and the intensity of polydipsic water drinking observed during the challenge session in individuals that had access to the water bottle actually emerged on the 8^th^ SIP session, namely when HD rats started to develop excessive, compulsive adjunctive drinking leading them to differ from LD rats (Fig. 4). This negative relationship was further shown to be primarily due to TH+ cells that did not co-express zif268 at the time of sacrifice (Fig. 4). Thus, the percentage of Arc+ cells that were also TH+ but zif268- tracked the emergence of compulsive adjunctive drinking behaviour following a pattern that was very similar, if not identical to that of the Arc mRNA copy number (Fig. 4).

The inverse relationship between Arc activation in the LC TH+ neurons and compulsivity was not observed for the immediate early gene zif268 or when rats did not have the opportunity to express their adjunctive behaviour, be it compulsive or not, during the challenge session (Fig. 4). What emerged in these individuals was a consistent positive relationship between the levels of drinking prior to the development of excessive adjunctive behaviour in vulnerable rats and activation of neurons in the LC, as shown by consistent positive correlations between water intake on baseline and early SIP sessions and the percentage of zif268+ cells, irrespective of the co-marker they express.

Further analyses confirmed that these relationships between polydipsic water drinking behaviour and Arc recruitment were specific to neurons as correlations were not observed consistently in GFAP+ cells (Fig. 4).

## Discussion

The results of the present study show that the development of compulsive adjunctive behaviour under SIP is associated with a decrease in Arc mRNA levels in the LC, and more particularly in a LC Arc+/TH+/zif268- neuronal ensemble. This new cellular signature of compulsive coping behaviour in the LC is in agreement with previous evidence of the involvement of this monoaminergic nucleus of the brainstem in stress [62] and coping strategies [61, 64], and in which originate the neurons whose projections to the NacS, further characterised in the present study, contribute to the regulation of the high impulsivity trait that confers and increased vulnerability to developing compulsive adjunctive behaviours under SIP [40, 59].

As previously shown [43, 100], the overall population of rats exposed to SIP progressively developed a polydipsic adjunctive response, which is accompanied by an activation of central dopaminergic and noradrenergic mechanisms [101]. However, individual differences emerged from the first week of training, with some individuals, namely HD rats, losing control over their coping response and developing excessive polydipsic drinking, the time course and magnitude of which is in line with that reported in previous studies from our laboratory and others [41, 43, 91, 102].

Like compulsions in humans with ICSDs, compulsive adjunctive responses in these HD rats produced a transient decrease in plasma corticosterone levels, in agreement with previous observations [21, 25, 26, 35] and those of a decrease in anxiety [40]. However this transient anxiolysis occurs in the context of a stress surfeit, which characterises compulsive disorders [103, 104] manifested in SIP as an overall heightened baseline level of anxiety [40] and plasma corticosterone [37].

The expression of these transiently stress-reducing compulsive responses was accompanied here by a decrease in Arc mRNA levels in the LC. Prevention of the expression of compulsive drinking at test, by removal of the water bottle, resulted in an increase in Arc mRNA levels which reached those of LD rats. The decrease in Arc mRNA levels that selectively accompanied the expression of compulsivity at test tracked its development more than 16 days beforehand, as revealed by the emergence of a systematic negative correlation between Arc mRNA levels and daily water intake on session 8, the time at which HD rats started to differ from LD rats in their expression of polydipsic drinking. Altogether these data suggest that downregulation of the activity of the immediate early gene Arc in LC, and especially in TH+ zif268- neurons, contributes to the loss of control over adjunctive polydipsic drinking, which underlies in HD rats the vulnerability to develop compulsive behaviours. In light of the delicate interplay that exists between LC function and glucocorticoids [47, 48, 105–107], the lower level of plasticity observed in this Arc-defined LC neuronal ensemble in HD rats may be related to the blunted response to stress they have been shown to display [108] akin to individuals with OCD [109].

In addition, these observations are in line with evidence for functional alterations of the noradrenergic system in patients with compulsive disorders including genetic polymorphisms in the COMT gene [110–113], involved in the break-down of NA, elevated plasma levels of NA metabolites [112] and altered neuroendocrine responses to adrenergic drug challenges [114, 115]. Similarly, chronic systemic administration of the NA reuptake blocker atomoxetine prior to exposure to SIP prevents the development of compulsive coping in highly impulsive rats [41] whereas it exacerbates the expression of compulsivity in HD rats when introduced after long-term exposure to SIP [116]. This suggests that the altered noradrenergic function that underlies impulsivity and the associated increased tendency to develop compulsive coping behaviours, which is damped by atomoxetine [41], mutates during the recruitment by SIP exposure of ascending dopaminergic and monoaminergic systems [101]. A potential mechanism is that atomoxetine, whose potentiation of extracellular levels of NA decreases spontaneous activity of LC neurons via α2-adrenoceptor stimulation in the LC [117], promotes a further decrease in the expression of Arc in the LC Arc+/TH+/zif268- neuronal ensemble in HD rats, thereby exacerbating their compulsivity.

At the neural systems level, as discussed earlier, the influence of atomoxetine on impulse control has been shown to be mediated by the medial portion of the NacS. Bilateral intracerebral infusions of atomoxetine into the NacS, but not into the Nucleus Accumbens Core replicated the effects of its systemic administration on premature responses in the 5-choice serial reaction time task [59]. This specific region of the NacS in which atomoxetine administration effectively reduces impulsivity receives noradrenergic innervation from the LC, as demonstrated in the present study by a much higher density of LC neurons labelled after infusions of the retrograde virus CAV2-GFP in that, as compared to, other territories of the NacS. Future research is warranted to determine whether the Arc+/TH+/zif268- LC neurons that display a specific decrease in Arc mRNA levels associated with the development of compulsive adjunctive behaviours are those that project to this region of the NacS [60, 118]. Further investigations are also warranted to determine whether a similar ensemble is recruited in the development of compulsive coping behaviour in females in whom the functional engagement of the LC in coping behaviours has been reported to differ from that of males [61, 62].

Nevertheless, the results of the present study shed new light on the cellular and molecular basis of the involvement of the LC in the development of compulsion resulting from the loss of control over coping strategies, one of the earliest evidence for which was the demonstration that bilateral lesions of the LC decrease water consumption in rats exposed to a SIP procedure without influencing homoeostatic thirst [66]. Since, it has been shown that the individual tendency to engage in active vs passive coping responses and the ensuing differential resistance to stress engages different adaptations in the neural circuits controlling the LC-NA stress response system [61] in a genetically determined manner [65].

The mechanisms by which downregulation of Arc in a TH+ zif268- neuronal ensemble in the LC accompanies or perhaps underlies the expression of compulsive adjunctive behaviours in vulnerable individuals remain unknown. Arc is broadly expressed at low levels under resting conditions and its transcription is rapidly and transiently induced following synaptic integration [119, 120]. Arc expression is regulated by emotionally relevant experiences, including stressful situations [75, 85] or alcohol withdrawal, in a large number of brain regions; including the basolateral amygdala [121] and the Nac in which a large increase in Arc mRNA and protein levels is triggered by exposure to social defeat stress [75]. Viral overexpression of Arc in the Nac of Arc- knockdown mice is sufficient to rescue anxiety-like behaviours [122], thereby demonstrating causally that striatal Arc contributes to the regulation of anxiety [122], supposedly through its influence over dendritic plasticity [85]. Indeed, Arc mRNA is trafficked to neuronal dendrites [123] and, induced by neuronal activity [124], translated into proteins that can promote both synaptic strengthening and weakening [125]. In agreement with these observations, Arc has been shown to be involved in multiple forms of glutamatergic plasticity [126–128]. Overexpression of Arc blocks the homoeostatic increase in AMPA-type glutamate receptors (AMPARs), whereas its decrement results in increased AMPAR function and a reduction of homoeostatic scaling of AMPARs [129]. Thus, Arc is well positioned to influence glutamate-dependent plasticity and its function in learning and memory [130]. In agreement with other clinical and preclinical studies having established the involvement of altered glutamatergic function in compulsive disorders, the tendency compulsively to express a polydipsic adjunctive behaviour under a SIP procedure has been shown to be associated with a lower level of glutamate in the medial prefrontal cortex [45], and reduced by glutamatergic drugs such as memantine [131]. Together with the previous evidence that compulsive symptoms are decreased by glutamatergic drugs in patients with ICSDs [132, 133], this observation suggests that the downregulation of Arc mRNA levels in the LC observed in vulnerable individuals when they express a compulsive adjunctive behaviour may be reflective of altered glutamatergic integration by LC neurons. Further research is warranted to establish which circuit, if any, among the glutamatergic inputs to the LC that include the paragigantocellularis nucleus, the lateral habenula and prefrontal cortex [134–136], are involved in these adaptations.

Within the LC, this study has revealed that a differential recruitment of Arc- vs zif268- functional ensembles is associated with resilience to the loss of control over coping strategies. Thus, rats which expressed a compulsive polydipsic response showed a specific downregulation of Arc mRNA in TH-positive neurons that did not co-express zif268. In contrast, the mRNA levels of each marker in cellular ensembles or cell types in which they were both expressed did not correlate with polydipsic drinking when it became compulsive. While little is known about the functional relationship between Arc and zif268, the transcription of the former has been shown to be under direct regulation of Egr family of transcription factors to which the latter belongs [137], thereby suggesting that the two factors should show converging mRNA levels, as they do in ensembles in which their mRNA levels do not correlate with compulsive drinking. It has indeed been shown that exposure to nicotine, for instance, results in converging increases in Arc and zif268 mRNA levels [88]. However, such studies relied on classical in situ hybridisation, which did not enable a multiplex approach necessary to determine whether the increases in mRNA levels were occurring in the same cell type or across different ensembles. In other studies on cortical and hippocampal neurons, it has been shown that while most of the cells that expressed Arc also expressed zif268, some zif268+ cells did not express Arc. This degree of independence between the functional recruitment of the two IEGs, which would be a prerequisite for the apparent higher sensitivity to behavioural demands that the effector IEG Arc shows as compared to other IEGs, including Zif268 [138] suggests that there may be regulatory mechanisms in addition to, and competing with, those involving zif268 in the control of Arc mRNA levels. Transcriptional activation of Arc by zif268 is indeed completely inhibited by coregulatory factors such as Nab2 [139]. Further research will be necessary to better understand the molecular mechanisms that contribute to the emergence of this Arc+/TH+/zif268- specific LC neuronal ensemble in which a downregulation of Arc selectively characterises the expression of compulsive adjunctive behaviour in vulnerable individuals.

Altogether, the findings of the present study identify an ensemble in the LC characterised by a decrease in the expression of Arc in TH+ zif268− neurons as a cellular marker of the expression of compulsive adjunctive drinking, thereby opening avenues for a mechanistic understanding of the role played by specific neuronal ensembles in the LC in coping behaviours and their compulsive manifestations.

## Supplementary information

Supplementary online methods
