# Supplementary material for: The development of compulsive coping behaviour is associated with a downregulation of Arc in a Locus Coeruleus neuronal ensemble
Source: Neuropsychopharmacology. 2023 Jan 12;48(4):653–63. doi: 10.1038/s41386-022-01522-y (PMC9938202; doi:10.1038/s41386-022-01522-y)
Supplement: Supplementary file 1 — Supplementary online methods [file 41386_2022_1522_MOESM1_ESM.docx]

**The development of compulsive coping behaviours is associated with a downregulation of Arc in a Locus Coeruleus neuronal ensemble**

# Authors:

Clara Velazquez-Sanchez^1^, Leila Muresan^2^, Lucia Marti-Prats^1^, and David Belin^1^

^1^CLIC (Cambridge Laboratory for research on Impulsive/Compulsive disorders), Department of Psychology, University of Cambridge, Downing Street, Cambridge CB2 3EB, UK.

^2^Cambridge Advanced Imaging Centre, Department of Physiology Development and Neuroscience of the University of Cambridge, Downing Street, Cambridge CB2 3DY, UK.

# Corresponding author:

Professor David Belin

Department of Psychology,
University of Cambridge
Downing St.
Cambridge CB2 3EB, UK
Tel. +44 (0) 1223 334016

email: bdb26@cam.ac.uk

# Supporting Online Materials

# Supporting online Materials and methods

## Timeline of the experiments.

As illustrated in **Figure 1**, one week of habituation to the animal facility, rats from the first experiment received intra-NacS infusion of a retrograde CAV2-GFP virus under stereotaxic surgery and were left undisturbed for at least one week. In order to establish for future experiments that such viral-mediated expression of transgenes did not interfere with the individual trajectories that are observed with regard to the development of adjunctive drinking under SIP, all rats were then tested in the exact same procedure as that used in the subsequent experiment. Thus, rats from both experiments were progressively food restricted to 80% of their theoretical free-feeding body weight. They were accustomed to the SIP context over two habituation sessions during which their regulatory water intake was measured and then trained in a SIP procedure for 21 daily sessions. Ninety minutes after the last SIP session, rats from experiment 1 underwent a blood collection, in order to assess post-SIP plasma corticosterone levels, after what they were perfused transcardiacally, and their brains harvested in order subsequently to map the projections of the LC to the NacS. In contrast, rats from experiment 2 were sacrificed forty-five minutes after a 60 min challenge session with or without the opportunity to express their adjunctive behavior, and their fresh brains were harvested and properly stored subsequently to be used for RNAscope assays.

## Apparatus

The SIP procedure was carried out as previously described [1,2] in 12 operant chambers located in ventilated and sound-attenuating cubicles (Med Associates, St. Albans, VT) controlled by MedPC software (Med Associates Inc., Ltd). Each operant chamber was made of aluminium and transparent acrylic plastic with a stainless-steel grid floor (24 x 25.4 x 26.7 cm) and was equipped with a house light (3-W), a food tray (magazine), installed at the centre of the front wall, and a bottle from which a stainless-steel sipper tube delivered water into a receptacle placed in a magazine on the wall opposite the food magazine.

## Schedule-induced polydipsia (SIP)

The day before the first habituation session, rats were exposed in their home cages to the same 45 mg food pellets used in the SIP procedure in order to avoid any neophobia. On a first habituation session, rats were exposed for one hour to the operant chambers and were given access to water and 60 food pellets (45mg, *TestDiet, USA*) that were previously placed in the food magazine. On the second 1-hour habituation session, rats were exposed to a random interval (RT-60 seconds) schedule of pellet food delivery in order to ensure their regulatory water intake was measured over a 60 min period while they learnt that food was being dispensed in the magazine.

The SIP procedure was based on fixed-time 60-second schedule of food delivery, conditions previously shown to result in marked individual differences in the propensity to develop compulsive adjunctive drinking behavior [1-4]. Twenty-four hours after the baseline session rats underwent 21 of these FT-60s SIP sessions [1,5]. Three hundred ml bottles were filled daily with fresh tap water, weighted, and placed into the operant boxes immediately before the start of each session. House lights were switched on at the beginning and switched off at the end of each session. The total volume of water consumed during the session was calculated daily as the difference between the bottle weight before and after the session.

## Experiment 1

### Stereotaxic surgery and viral infusions

In order to identify which territory of the LC projects to the area of the NacS in which infusions of atomoxetine recapitulate the effect of systemic administration of impulse control [6] intra-NacS infusions of a CAV2-GFP virus were carried out unilaterally at 4 different anteroposterior coordinates into that structure (**Figure 3)** using a stereotaxic frame (WPI Hitchin, UK) under isoflurane anaesthesia (O2: 2 L/min; 5% for induction and 2-3% for maintenance) and analgesia (Metacam, 1 mg/kg, sc., Boehringer Ingelheim). The analgesic treatment was continued orally for three days post-surgery. The forty-eight rats were divided into four groups, each receiving a unilateral CAV2-Cre virus (109vp/μl, 1μl/side) infusion at the following stereotaxic coordinates AP: +2.76, +2.28, +1.7 or +1.08; ML: ±1.0, ±1.0, ±0.8, ±1.0; DV: −6.8, −7.2, −7.25, −7.1, respectively (from the skull) [7]. Infusions were performed at a rate of 0.15 μl/min with 10ul Hamilton syringes placed in a Harvard infusion pump and connected with a polyethylene tubing to 24-gauge injectors (Coopers needle works Ltd). Injectors were left in place for 7 minutes after the infusion to allow for diffusion. Animals were sacrificed at least 2 months after the viral infusion, so that the retrograde virus had time to travel from the injection site to the LC.

### Histology

## Animals were euthanized with an overdose of sodium pentobarbital (300 mg; Dolethal; Vétoquinol UK Ltd, Buckingham, UK) and transcardiacally perfused with isotonic saline followed by 4% neutral buffered formalin (NBF). Brains were collected and kept in 4% NBF for an additional 24h at 4°C, after what they were transferred into a 30% sucrose solution in 0.01 M PBS until saturation. After a quick freezing on dry ice, brains were sectioned into 30 μm thick coronal sections using a cryostat (Leica CM3050 S Research Cryostat) and stored in a cryoprotectant solution at -20°C until being processed for immunofluorescence.

## GFP immunofluorescence

Every sixth section, ranging from -9.6 to -9.96 mm from bregma, a rostrocaudal region that entirely encompasses the LC was processed for immunofluorescence. Briefly, free-floating sections were washed 3 x 10 min in a phosphate saline (PBS) buffer at room temperature. Sections were then blocked for 2h in 5% bovine serum albumin (BSA, Sigma-Aldrich, A7906) in 0.01M PBS and 0.3% Triton X-100 (Sigma-Aldrich, T8787) prior to being incubated with the primary antibody (chicken anti-GFP; 1:1000; abcam, ab13970) in 2% BSA and 0.1% Triton X-100 overnight (18h) at 4°C. Sections were then washed for then minutes in 0.01M PBS three times and incubated in the secondary antibody (goat Anti-chicken AF488, 1:1000; ThermoFisher Scientific, A-11039) for 2h at room temperature. Sections were again washed 3 x 10 min with 0.01M PBS, mounted onto glass slides (Fisherbrand Superfrost Microscope Slides) and allowed to dry overnight (protected from light). Slides were then covered with a coverslip and fluoroshield mounting medium (abcam, ab104135) and stored at 4°C prior to image acquisition.

Images were acquired with a Zeiss Axio Imager M2 equipped with an AxioCam MRm camera (Oberkochen, Germany) using Visiopharm® software (Medicon Valley, Denmark), either at magnification 5x and tiled to create the whole slice images or at magnification 10x for a detailed analysis of the region of interest, namely the LC.

## Corticosterone Assay

Ninety minutes after the last SIP session, immediately prior to perfusion, blood was extracted directly from the heart of each of the rats involved in experiment 1 and collected in tubes containing K3 EDTA (Greiner Bio-One). Blood was centrifuged at 3000 r.p.m. for 10 min at 4°C (Eppendorf, centrifuge 5418R). The plasma was collected in new eppendorfs and stored at -80°C until assayed. Plasma levels of corticosterone were determined by ELISA according to the manufacturer’s kit advice (Cayman Chemical, 501320). All samples were measured in duplicate. The detection limit of the kit is 24 ng/ml. The antibody in the kit specifically reacts with corticosterone and has less than 1% cross-reactivity with other adrenal hormones (i.e. aldosterone and cortisone). The product of the enzymatic reaction was determined spectrophometrically at a wavelength between 405-420 nm using a plate reader (Azure Ao Absorbance Microplate Reader). Corticosterone concentrations (ng/ml) were determined by comparing samples to the standard curve generated.

## Experiment 2

### Histology

45-min after the challenge session, animals were briefly anaesthetized with isoflurane (<30s), decapitated and their fresh brains harvested, snap frozen at -40°C in isopentane (Sigma-Aldrich) and stored at -80°C, as previously described [8]. Brains were then processed using a cryostat (Leica Microsystems) into 12 μm thick coronal sections collected on Superfrost gelatine-coated slides (Fisher Scientific) and stored at -80°C until they were processed for multiplex RNAscope® in situ hybridization.

### RNAscope® in situ hybridization assay

RNAscope was performed according to the manufacturer’s instructions for fresh frozen tissue using the RNAscope Multiplex Fluorescent Reagent Kit (Advanced Cell Diagnostics). Brain sections were first fixed in chilled 10% NBF for 30 min on ice, rinsed 3 times in PBS and dehydrated in increasing concentrations of ethanol (50, 70, 100 and 100%). Slides were then kept in fresh 100% ethanol at -20°C overnight. The following day, sections were air-dried and heated at 37°C on a hot plate for 20 min to prevent their detachment from the slides. Next, a hydrophobic barrier was drawn around each section to avoid the loss of the reagents during the assay. Sections were treated with Protease IV for 20 min at room temperature and then rinsed 3 x 5 min with PBS prior to being incubated with the target probes in a HybEZ oven for 2h at 40°C.

Each probe consists of a unique oligonucleotide mixture designed to bind to a specific target RNA, which, for this study, was Early Growth Response 1 (Egr1/zif268 probe) [GenBank accession number NM_012551.2, target nt region 162-1333], Tyrosine Hydroxylase (TH probe) [GenBank accession number NM_012740.3, target nt region 422-1403], Glial Fibrillary Acidic Protein (GFAP probe) [GenBank accession number NM_017009.2, target nt region 1539-2534], Activity-Regulated Cytoskeleton-Associated Protein (Arc probe) [GenBank accession number NM_019361.1, target nt region 269-1148]. Due to a limitation to 3 fluorescence microscopy filters, two different complementary assays were performed combining the target probes with different colour channels as follows: Egr1/zif268 (channel1)-TH (channel 2)-Arc (Channel4), and Egr1/zif268 (channel1)-GFAP (channel 2)-Arc (Channel4).

Following the 2h-incubation with the target probes, sections were incubated with the preamplifier and amplifier probes (AMP1, 40°C for 30 min; AMP2, 40°C for 15 min; AMP3, 40°C for 30 min) and washed with washing buffer in between each incubation step for 3x 5min. Then sections were incubated with the fluorescence labelled probe (AMP4 AltB-FL) to detect the three triple combination channels in orange (Alexa Fluor 550 nm), green (Alexa Fluor 488 nm) and far red (Alexa Fluor 647 nm), respectively. The sections were rinsed in washing buffer and incubated with DAPI for 20s before being coverslipped with Fluoroshield mounting medium (Abcam, ab104135).

# Supporting online references

1 Ansquer S, Belin-Rauscent A, Dugast E, Duran T, Benatru I, Mar AC, et al. Atomoxetine decreases vulnerability to develop compulsivity in high impulsive rats. Biol Psychiatry. 2014;75(10):825-32.

2 Fouyssac M, Puaud M, Ducret E, Marti-Prats L, Vanhille N, Ansquer S, et al. Environment-dependent behavioral traits and experiential factors shape addiction vulnerability. Eur J Neurosci. 2020.

3 Pellon R, Ruiz A, Moreno M, Claro F, Ambrosio E, Flores P. Individual differences in schedule-induced polydipsia: neuroanatomical dopamine divergences. Behav Brain Res. 2011;217(1):195-201.

4 Moreno M, Flores P. Schedule-induced polydipsia as a model of compulsive behavior: neuropharmacological and neuroendocrine bases. Psychopharmacology (Berl). 2012;219(2):647-59.

5 Lopez-Grancha M, Lopez-Crespo G, Sanchez-Amate MC, Flores P. Individual differences in schedule-induced polydipsia and the role of gabaergic and dopaminergic systems. Psychopharmacology (Berl). 2008;197(3):487-98.

6 Economidou D, Theobald DE, Robbins TW, Everitt BJ, Dalley JW. Norepinephrine and dopamine modulate impulsivity on the five-choice serial reaction time task through opponent actions in the shell and core sub-regions of the nucleus accumbens. Neuropsychopharmacology. 2012;37(9):2057-66.

7 Paxinos G, Watson C. The rat brain in stereotaxic coordinates, 7th ed*.* 7th ed. Elesvier Academic Press: San Diego; 2013.

8 Fouyssac M, Pena-Oliver Y, Puaud M, Lim NTY, Giuliano C, Everitt BJ, et al. Negative Urgency Exacerbates Relapse to Cocaine Seeking After Abstinence. Biol Psychiatry. 2022;91(12):1051-60.
